# Effect of invasive acupuncture on awakening quality after general anesthesia: systematic review and meta-analysis

**DOI:** 10.3389/fmed.2024.1502619

**Published:** 2025-01-13

**Authors:** Fan-dong Bu, Shang-kun Si, Dong-bin Zhang, Yong-liang Chi

**Affiliations:** ^1^The First Clinical College of Shandong University of Traditional Chinese Medicine, Jinan, China; ^2^First Teaching Hospital of Tianjin University of Traditional Chinese Medicine, Tianjin, China; ^3^National Clinical Research Center for Chinese Medicine Acupuncture and Moxibustion, Tianjin, China; ^4^Tianjin University of Traditional Chinese Medicine, Tianjin, China; ^5^Department of Anesthesiology, Affiliated Hospital of Shandong University of Traditional Chinese Medicine, Jinan, China

**Keywords:** invasive acupuncture, general anesthesia, awakening, systematic review, meta-analysis

## Abstract

**Background:**

The process of waking up from general anesthesia is still not well understood, and recovery issues such as delayed awakening, agitation, postoperative cognitive dysfunction, continue to be a challenge for anesthesiologists. Currently, the treatment of these complications is mainly achieved through the antagonistic action of specific drugs, but sometimes the antagonistic drugs are not as effective as they should be and can add to the financial burden of the patient. Acupuncture, a common treatment in Traditional Chinese Medicine, is widely used around surgery. However, there is no enough evidence to show it improves recovery after anesthesia. To explore this, we reviewed relevant randomized trials and conducted a meta-analysis.

**Objective:**

This systematic review was conducted to explore the effect of perioperative application of invasive acupuncture on the quality of postoperative awakening after general anesthesia.

**Methods:**

By searching PubMed, Embase, Cochrane Clinical Trials Center, China Knowledge Network (CNKI), China Biomedical Database (CBM), Wanfang Medical Database, Weipu Database, to include randomized controlled trials of invasive acupuncture applied perioperatively. Search is limited from the build-up of the database to March 2022. The statistical analysis was conducted using RevMan 5.3. Quality assessment of the included research literature using Cochrane-recommended risk of bias assessment tool.

**Results:**

18 randomized controlled trials were included with 1,127 patients. 565 patients in invasive acupuncture intervention group, 562 patients in control group. Results showed that invasive acupuncture group had a shorter eye opening time than control group (MD = −6.42, 95% CI [−8.17, −4.66], *p* < 0.001), shorter extubation times (MD = -5.84, 95% CI [−8.12, −3.56], *p* < 0.001), lower MAP at extubation (MD = −18.54, 95% CI [−22.69, −14.39], *p* < 0.001), lower HR at extubation (MD = −14.85, 95% CI [−23.90, −5.81], *p* < 0.001). No statistical difference in the occurrence of POCD (OR = 0.56, 95% CI [0.28, 1.11], *p* = 0.10) and postoperative agitation (OR = 0.42, 95% CI [0.11, 1.65], *p* = 0.21).

**Systematic review registration:**

https://www.crd.york.ac.uk/PROSPERO/, CRD42023410260.

## 1 Introduction

The period of awakening after general anesthesia is both crucial and potentially hazardous (1). Complications such as delayed awakening, emergence agitation (EA), and cardiovascular issues can increase the risk of patient harm. Reports indicate that approximately 19% of adults experience EA following non-cardiac surgeries (2), EA is typically characterized by irritability, purposeless movements, and heightened arousal during the early recovery phase from anesthesia (3). Unlike postoperative delirium (POD), EA involves aggressive behaviors that can pose significant risks to healthcare professionals (4). The process of awakening includes the removal of the tracheal tube, which can activate the sympathetic catecholamine system, leading to increased oxygen consumption and the potential for brain damage (1). Current strategies for managing adverse reactions during this period primarily involve drug antagonism and enhanced analgesia. However, the administration of antagonistic drugs may result in additional complications, such as increased sedation during surgery in patients with EA, which could cause delays in awakening.

Invasive acupuncture, which includes techniques such as hand-twisting and electroacupuncture, is rooted in traditional Chinese medicine. It is thought to regulate the balance between the sympathetic and parasympathetic nervous systems and has demonstrated anti-inflammatory effects through various signaling pathways (5, 6). Owing to its safety, convenience, and fewer side effects, invasive acupuncture is commonly used in clinical settings (7–9).

While invasive acupuncture is commonly employed, there is still a considerable gap in meta-analyses examining its effects on the quality of recovery from general anesthesia. Thus, this study aims to perform a meta-analysis of randomized controlled trials (RCTs) to investigate if invasive acupuncture can affect the quality of awakening. The ultimate goal is to provide an evidence-based framework for clinical decision-making aimed at improving recovery quality during the awakening phase and providing higher-quality evidence.

In this research, invasive acupuncture is defined as including both electroacupuncture and traditional acupuncture, with the intervention group receiving one of these treatments. The time to eye opening is defined as the period from the end of the procedure until the patient opens their eyes, while the time to extubation is defined as the interval from the end of the procedure until the endotracheal tube is removed. Postoperative cognitive dysfunction (POCD) was defined as a decrease of one standard deviation in the Mini-Mental State Examination (MMSE) score at 3 days postoperatively compared to the baseline score obtained 1 day preoperatively. The control group was given either a blank control or a sham intervention.

### 1.1 Objectives

The aim of this study was to assess the impact of the application of invasive acupuncture on the quality of awakening in postoperative patients undergoing general anesthesia.

## 2 Methods

### 2.1 Inclusion criteria

(1) Study type: RCT including the effect of invasive acupuncture (Electroacupuncture, acupuncture) on the quality of postoperative awakening after general anesthesia; language is not limited. (2) Study population: The study included patients who underwent surgery under general anesthesia and received either invasive acupuncture or blank/sham stimulation during the perioperative period. No limitations were placed on age, gender, or nationality. (3) Interventions: Invasive acupuncture group received electroacupuncture, or acupuncture. The control group was not subjected to acupuncture stimulation, nor did they receive stimulation at non-meridian or non-acupuncture points.

### 2.2 Exclusion criteria

(1) Studies involving patients undergoing surgeries without general anesthesia or receiving other interventions were included in the analysis. (2) Original text was inaccessible or if the outcome indicators were incomplete. (3) Studies from non-RCTs, systematic evaluations or reviews, mechanistic studies, conferences and animal trials.

### 2.3 Information sources

BF and SS searched Chinese and English databases on 2022/03/24, included PubMed, Embase, Cochrane Clinical Trials Center, Chinese National Knowledge Infrastructure (CNKI), Wei-pu Database (VIP), Chinese Biomedical Database (CBM), Wan-fang Database, and article were searched from the time of database creation to March 2022.

### 2.4 Search strategy

The Chinese search terms included: “general anesthesia,” “awakening,” “recovery of consciousness,” “open eyes,” “extubation,” “post-anesthesia monitoring treatment room,” “acupoint “, “acupuncture,” “electroacupuncture,” “needle acupuncture,” “randomized controlled trial.” English search terms included: “general anesthesia,” “awakening,” “emergence,” “recovery,” “eye-opening,” “extubation,” “PACU,” “acupoint,” “acupuncture,” “electroacupuncture,” “randomized controlled trial.” The formula can be viewed in the Supplementary material S1.

### 2.5 Selection procedures

After de-duplication, two people initially screened by looking at titles and abstracts, and then they reviewed the full article. Key data, such as author names, publication years, sample sizes, interventions, and outcome indicators, were extracted from the final set of screened studies for analysis. If there was any disagreement, the corresponding author was consulted for resolution.

### 2.6 Data collection and data items

The data of the study was extracted by two independent persons (BF, SS) and the extracted data was saved in an Excel sheet. When there were doubts about the data in the study, they negotiated to resolve them. If data were not available for the outcome of interest, we contacted the authors of the data for information. The names and definitions of the extracted data are given in Supplementary material S2.

### 2.7 Study risk of bias assessment

Two researchers independently assessed the quality of the studies included in the review using a specific tool and criteria recommended by the Cochrane systematic review manual. The assessment focused on key elements including randomization, blinding, incomplete outcome data, and other potential sources of bias. The findings were classified into three categories: “low risk”, “high risk”, or “unclear”. Methodological and qualitative evaluation of the included literature was as follows. If a study lacks relevant information, the two individuals will contact the corresponding author of the relevant study to enquire. GRADE was also applied to assess the quality of evidence.

### 2.8 Outcomes

Time to open eyes, Time to extubation, MAP immediately after extubation, HR immediately after extubation is a continuous variable are continuous variables, statistical description and effect sizes were combined using Mean difference (MD), along with their corresponding 95% confidence interval (CI). Incidence of POCD and Agitation are dichotomous variables, statistical description and effect sizes were combined using Odds ratio (OR), along with their corresponding 95% confidence interval (CI).

### 2.9 Data synthesis

Review Manager 5.3 was applied for data analysis. All outcomes were analyzed using an intention-to-treat analysis. We evaluated the results using MD, OR values, and their 95% CI. A *p*-value of less than 0.05 was regarded as indicative of a statistically significant difference. Heterogeneity was evaluated using the chi-square test with a significance threshold of *α* = 0.1, and the extent of heterogeneity was quantified by the I^2^ statistic. When heterogeneity is obvious (I^2^ ≥ 50%), random effects model is used. When heterogeneity is low (I^2^ < 50%), using fixed effects model. The results of each synthesis are presented in the form of a forest plot. Funnel plots were used to evaluate publication bias when a sufficient number (n ≥ 10) of studies were available for analysis. A balanced distribution of points on the center line was considered a low level of publication bias. Egger’s test was employed to evaluate the presence of publication bias when there was a lack of sufficient study (*n* < 10). *p* < 0.05 suggests a publication bias.

### 2.10 Subgroup analysis

Based on age classification, we performed subgroup analyses of eye-opening time. One group is age >60, another group is age ≤ 60. Based on the type of surgery, we performed subgroup analyses of time to extubation. One group is minor surgery and one group is major surgery.

### 2.11 Sensitivity analysis

When I^2^ ≥ 50%, a sensitivity analysis of the literature was performed, the sensitivity analysis was done by excluding studies where the MD deviated more from the center in the forest plot produced by Review manager 5.3. A larger deviation means that more heterogeneity is likely to be produced. Re-perform sensitivity analysis until I^2^ is stable.

## 3 Results

### 3.1 Eligible studies and study characteristics

**Figure 1:**
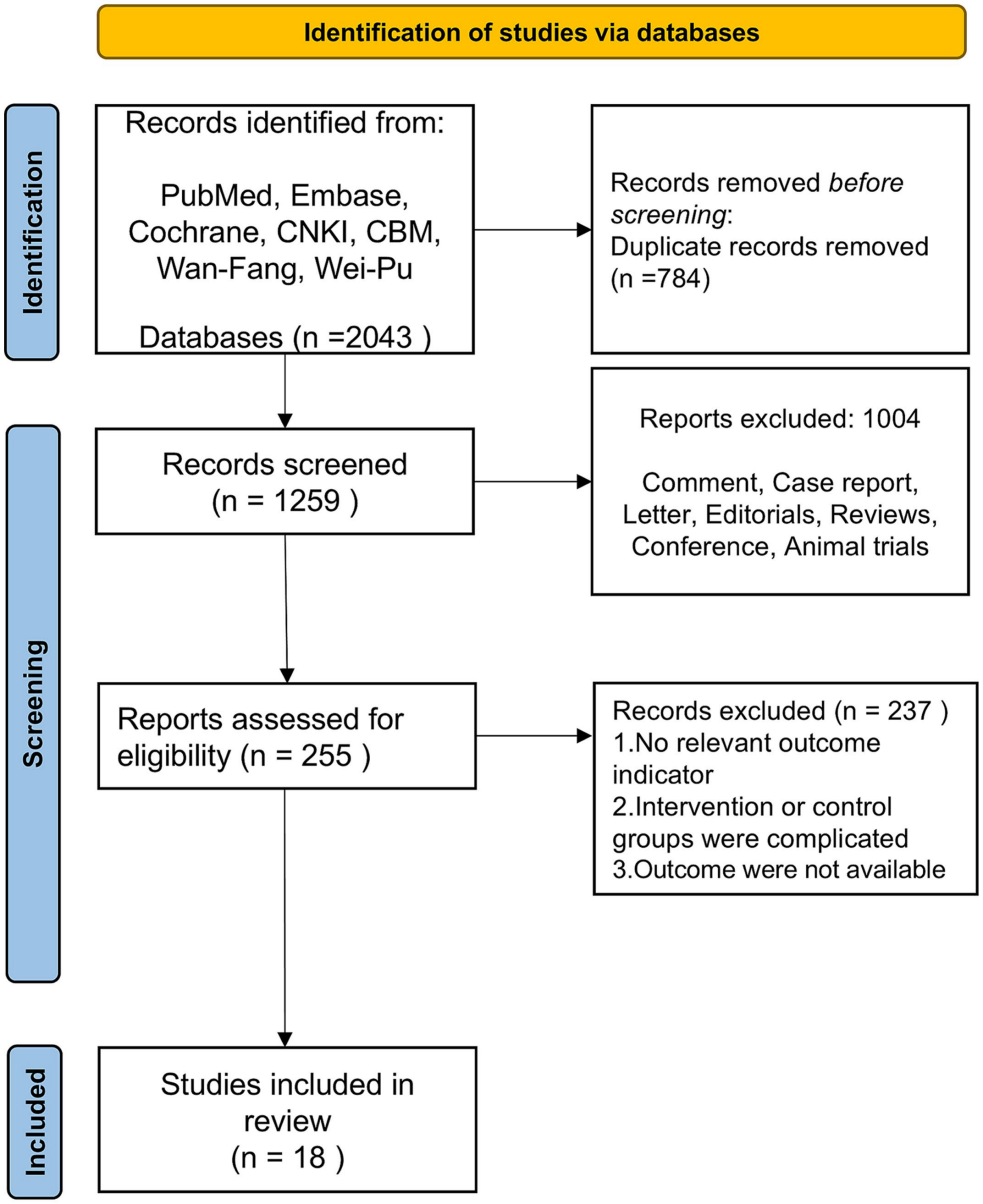
Flow chart of literature screening.

A total of 2,043 relevant literature was obtained from the search, 784 duplicates were excluded, 1,004 were excluded based on the title and abstract, After the full-text assessment, 18 studies meeting the study requirements were included, involved a total of 1,127 patients, 565 cases in invasive acupuncture group and 562 cases in control group. Figure 1 illustrates the literature screening process, Tables 1, 2 display the study characteristics and interventions details of the included studies. Good results in quality grading of evidence from included studies (see Supplementary material S3 GRADE).

**Table 1:** Study characteristics.

Authors, year	Country	Surgery	Anesthesia	Patient	Sample size		Outcome
					T	C	
Lu et al. (18)	China	PB	TIVA	Adult	30	30	①
Li et al. (19)	China	TEC	GA	Adult	30	30	③④
Yan et al. (20)	China	RREC	GA	Adult	20	20	②③④
Zheng et al. (21)	China	PE	TIVA	Adult	40	40	①
Liu et al. (22)	China	GTR	GA	Adult	30	30	①
Wang et al. (23)	China	LC	GA	Adult	30	30	②
Gemma et al. (24)	Italy	PAGA	GA	Adult	10	9	①②
Yang et al. (25)	China	LC	GA	Adult	50	50	②
Lin et al. (26)	China	GTR	GA	The aged	42	41	①
Yan et al. (27)	China	PN	GA	Adult	40	40	①②
Yu et al. (28)	China	UL	GA	Adult	20	20	①
Lin et al. (29)	China	BCR	GA	The aged	38	37	①
Yang et al. (30)	China	GL	GA	AF	30	30	①②③④
Fu et al. (31)	China	P	GA	Adult	12	12	④
An et al. (32)	China	STR	GA	Adult	40	40	①②
An et al. (33)	China	STR	GA	Adult	40	40	①②
Gu et al. (34)	China	C	GA	Adult	30	30	①②
Gemma et al. (35)	Italy	M	GA	Adult	33	33	②

AF, Adult females; TIVA, Total intravenous anesthesia; GA, general anesthesia; PB, Painless Bronchoscope; TEC, Thoracotomy for esophageal cancer; RREC, Radical resectionn of esophageal carcinoma; PE, Painless enteroscopy; GTR, Gastrointestinal tumor resection; LC, Laparoscopic cholecystectomy; PAGA, Patients after general anesthesia; PN, Percutaneous nephrolithotomy; UL, Unilateral lobectomy; BCR, Bowel cancer resection; GL, Gynecologic laparoscopy; P, Pneumonectomy; STR, Supratentorial tumor resection; C, Cholecystectomy; M, Microdiscectomy. ①: Time to open eyes; ②: Time to extubation; ③: MAP(immediately after extubation); ④:HR(immediately after extubation); ⑤:Adverse reaction including POCD and agitation.

**Table 2:** Details of interventions.

Authors, year	Operating model	Time point	Frequency (Hz)	Current (mA)	Acupoint
Lu et al. (18)	Acupuncture	30min before bronchoscopy	NA	NA	Bil (Area of 1,2,3)
Li et al. (19)	Electroacupuncture	30 min before the induction till the end	2	Unknown	Bil (SI3, SJ6, PC6, LI4)
Yan et al. (20)	Electroacupuncture	30 min before the induction till the end	Unknown	Unknown	Bil (Internal pitting, PC6)
Zheng et al. (21)	Electroacupuncture	30min before the induction till the end	2/100	Unknown	Until (ST36, GB34, ST37, SP6), Bil (Li4)
Liu et al. (22)	Electroacupuncture	30 min before the induction till the end of surgery	2/100	5~(X-1)	Bil (ST36, SP6)
Wang et al. (23)	Electroacupuncture	20min before the induction	30	Unknown	Bil (GB24, ST30, GB34)
Gemma et al. (24)	Acupuncture	After the surgery	NA	NA	Bil (KI1, DU6)
Yang et al. (25)	Acupuncture	10-20min before anesthesia induction, rotation stimulation every 10 minutes after injection until the end of the operation	NA	NA	Bil (LI4, LR3)
Lin et al. (26)	Electroacupuncture	30 min before the induction till the end of surgery	2/100	7~7.5	Bil (DU20, PC6, ST36)
Yan et al. (27)	Electroacupuncture	20 min before the induction till the end of surgery	2/100	7~7.5	Bil (PC6, Internal pitting, LI4, LI5RN3, ST30, LR10)
Yu et al. (28)	Electroacupuncture	30 min before the induction till the end of surgery	2/100	Unknown	Bil (PC6, LI3, SJ6, LI4)
Lin et al. (29)	Electroacupuncture	20 min before the induction till the end of surgery	4/20	7~7.5	Uk (DU20, PC6, ST36, SP6)
Yang et al. (30)	Electroacupuncture	20~30 min before the induction till the end of surgery	2/100	12~15	Bil (ST36, SP6, LI4, LR3)
Fu et al. (31)	Electroacupuncture	30 min before induction	Unknown	1-3	Bil (SI3, SJ6, LI4)
An et al. (32)	Electroacupuncture	From anesthesia beginning to the end of operation	2/100	Unknown	Uk (LI4, TE5, BL63, LR3, ST36, GB40)
An et al. (33)	Electroacupuncture	From the beginning of anesthesia induction to the end of surgery	2/100	0.3~2	Bil (GB20, BL2)
Gu et al. (34)	Electroacupuncture	15~30 min before the induction	4/20	X~5	Bil (LI4, PC6, ST36, GB34)
Gemma et al. (35)	Acupuncture	Unknown	NA	NA	Bil (K1, S36)

Bil, bilateral; Unil, unilateral; NA, not applicable. Uk (unknown): It was not clear whether it was unilateral or bilateral. Electroacupuncture: A needle that can be connected to a nerve stimulator for electrical stimulation. Acupuncture: Needles that require constant hand twisting to cause stimulation of acupuncture points. X: The intensity of the current that the patient has difficulty tolerating while no pain and other adverse reactions.

### 3.2 Bias assessment results

**Figure 2:**
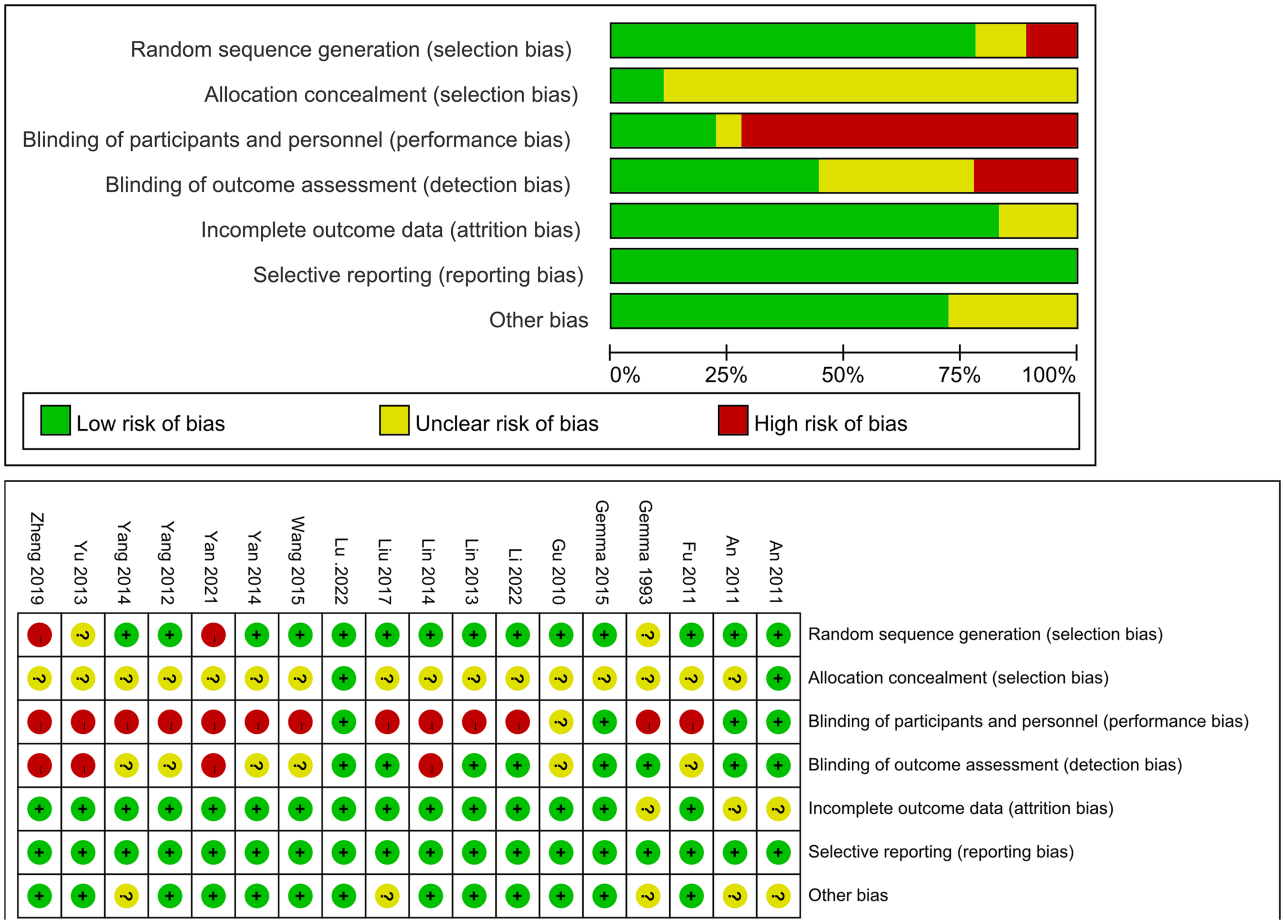
Risk of bias graph and risk of bias summary.

The assessment of bias for the included studies is shown in Figure 2. The overall risk of bias of the included studies showed good. Results of publication bias test in Supplementary material S4.

### 3.3 Effect of invasive acupuncture on eye opening time

**Figure 3:**
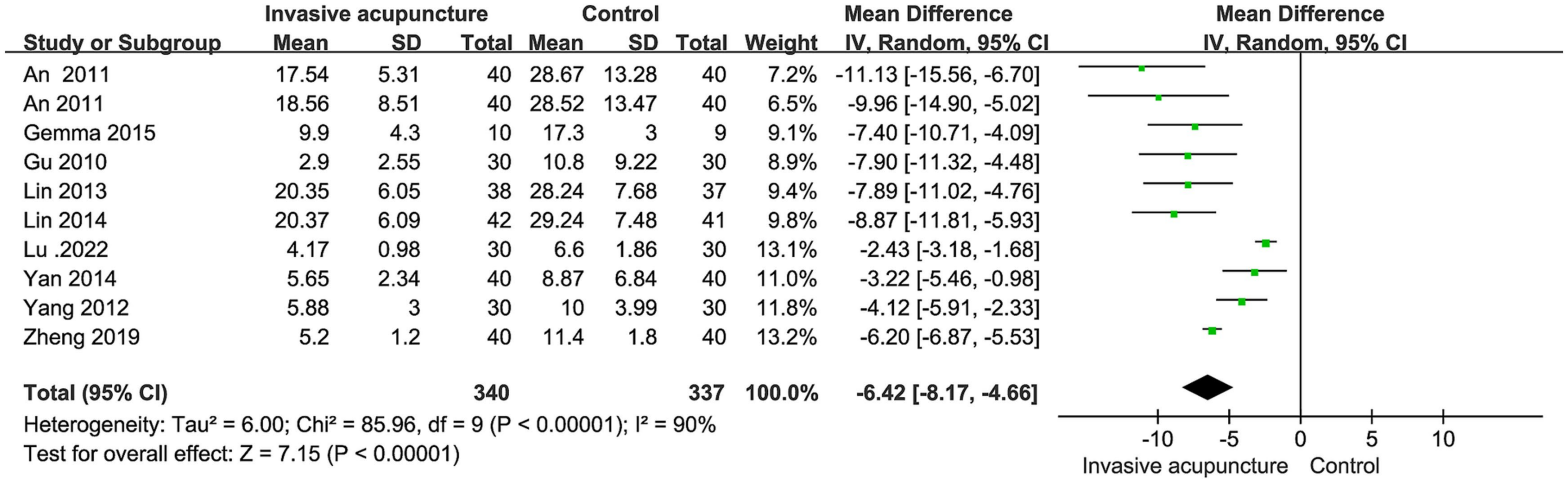
Synthesized results of eye-opening time.

10 studies mentioned time to open eyes. Included 677 patients, 340 in invasive acupuncture group, 337 in control group. Heterogeneity test I^2^ = 90% and random effects model was used to synthesize the data. Result shows that invasive acupuncture group had a shorter eye-opening time than control group (MD = −6.42, 95% CI [−8.17, −4.66], *p* < 0.001), as shown in Figure 3.

### 3.4 Effect of invasive acupuncture on extubation time

**Figure 4:**
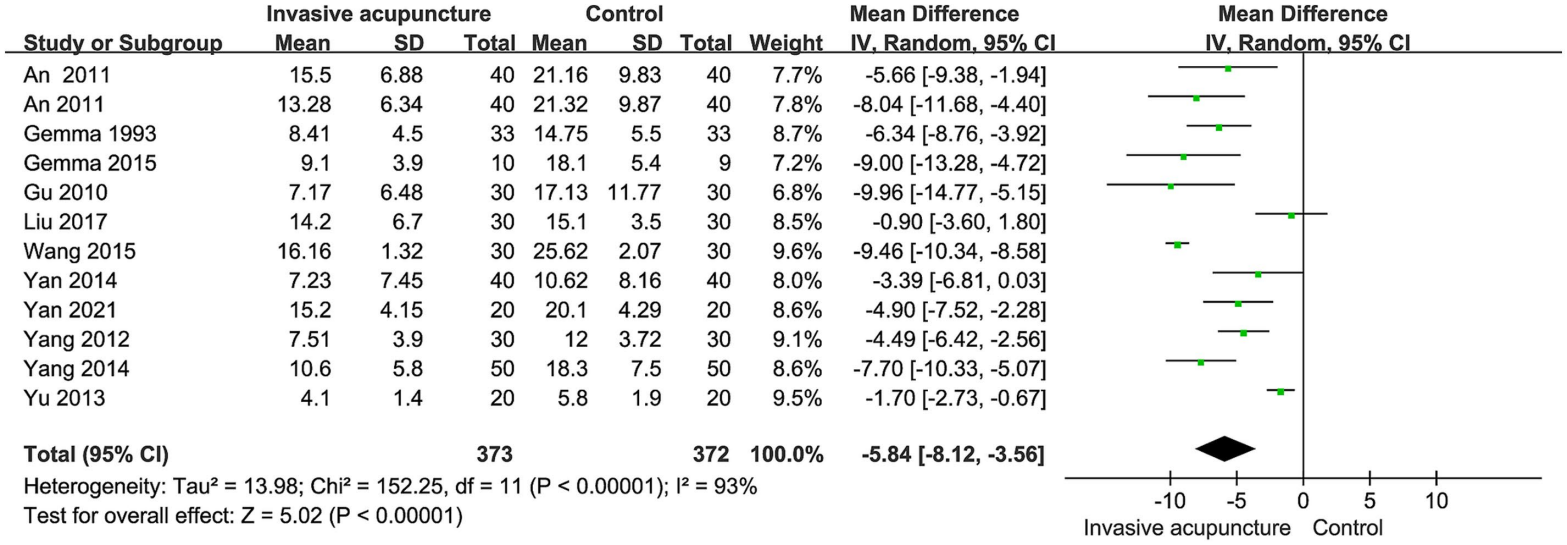
Synthesized results of extubation time.

12 studies mentioned the time to extubation. Included 745 patients, 373 in invasive acupuncture group, 372 in control group. I^2^ = 93%, with high heterogeneity, random effects model data synthesis results show that invasive acupuncture group has a shorter extubation time compared to control group (MD = -5.84, 95% CI [−8.12, −3.56], *p* < 0.001), as illustrated in Figure 4.

### 3.5 Effect of invasive acupuncture on MAP

**Figure 5:**
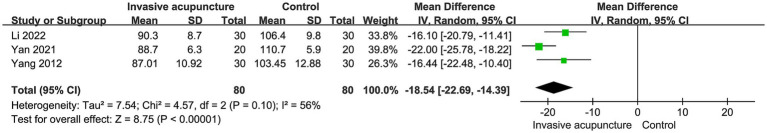
Synthesized results of MAP.

3 studies mentioned the mean arterial pressure (MAP) immediately following extubation. 160 patients in total, 80 in invasive acupuncture group, 80 in control group. Heterogeneity test I^2^ = 56%, using random effects model for combining effect sizes. The MAP was lower in invasive acupuncture group than in the control group (MD = −18.54, 95% CI [−22.69, −14.39], *p* < 0.001), as shown in Figure 5.

### 3.6 Effect of invasive acupuncture on HR

**Figure 6:**
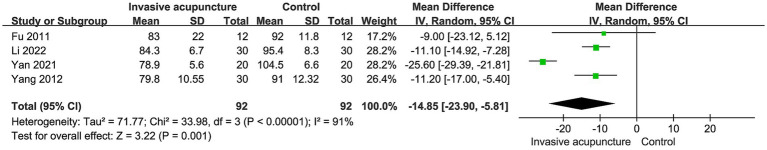
Synthesized results of HR.

4 studies mentioned heart rate (HR) at extubation. The synthesis included 184 patients, with 92 in the invasive acupuncture group and 92 in the control group. A heterogeneity test showed an I^2^ value of 91%, and the results were analyzed using a random effects model. Showed that HR was lower in invasive acupuncture group at the moment of extubation (MD = −14.85, 95% CI [−23.90, −5.81], p < 0.001), forest plot in Figure 6.

### 3.7 Incidence of POCD and agitation

**Figure 7:**
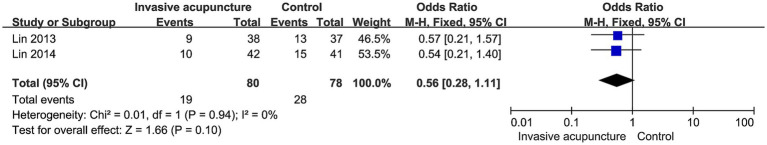
Synthesized results of POCD.

2 studies reported about the incidence of postoperative cognitive dysfunction (POCD). Included 158 patients, 80 in invasive acupuncture group while 78 patients in the control group. I^2^ = 0%, a fixed-effects model was used, the result showed that no statistical difference between invasive acupuncture group and control group on incidence of POCD (OR = 0.56, 95% CI [0.28, 1.11], *p* = 0.10), forest plot in Figure 7.

**Figure 8:**
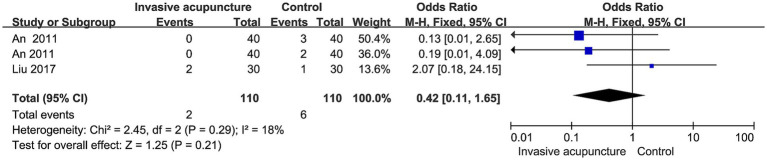
Synthesized results of agitation.

Three studies mentioned the incidence of postoperative agitation. 220 patients involved, 110 in invasive acupuncture group, 110 in control group. Heterogeneity was low (I^2^ = 18%), according to fixed effects model and effect size analysis no statistical difference was found on postoperative agitation (OR = 0.42, 95% CI [0.11, 1.65], *p* = 0.21), forest plot in Figure 8.

### 3.8 Subgroup analysis results

**Figure 9:**
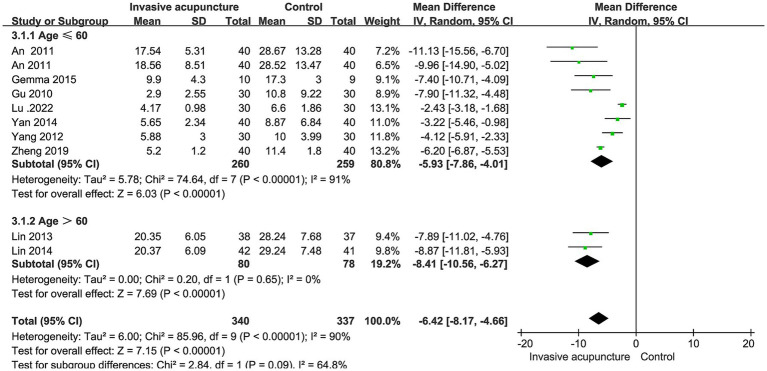
Subgroup analysis results of eye-opening time.

**Figure 10:**
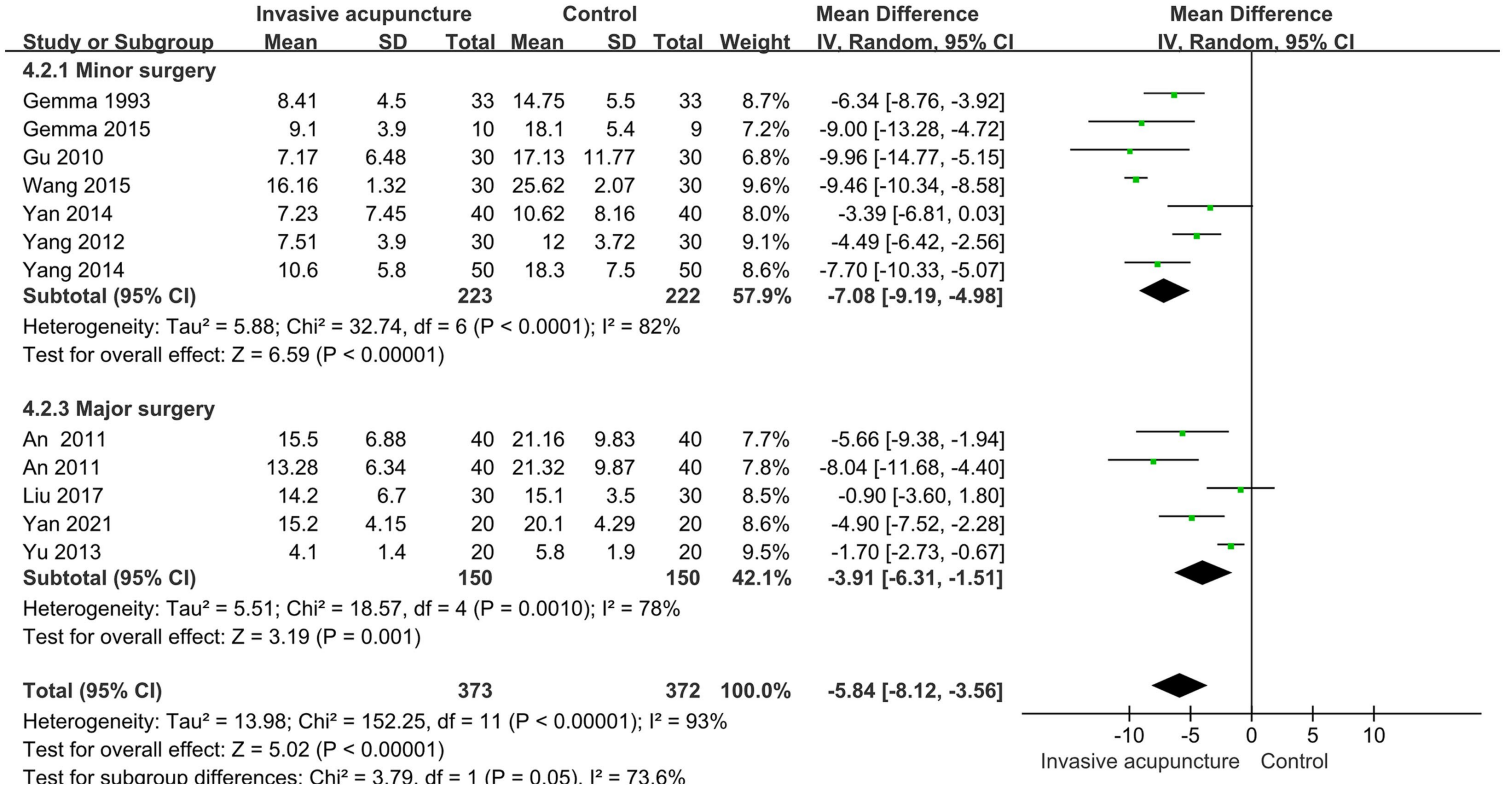
Subgroup analysis results of extubation time.

Based on age classification, we performed subgroup analyses of eye-opening time. One group is age >60, another group is age ≤ 60. Results suggests that heterogeneity may come from group age ≤ 60 (I^2^ = 91%). We performed subgroup analyses of time to extubation stratified by type of surgery (minor or major). The findings from these analyses suggest that the type of surgery does not appear to be a significant source of heterogeneity in the synthesized results for time to extubation, as shown in Figures 9, 10.

### 3.9 Sensitivity analysis results

For single studies with large heterogeneity, excluding any one evidence in Revman 5.3 had no effect on the overall effect. This suggests that the results of our study are robust and reliable. In the synthesized analysis of immediate heart rate at extubation, we observed that Yan 2021 has a great heterogeneity.

## 4 Discussion

All synthesized results show that perioperative application of invasive acupuncture accelerates awakening from general anesthesia and improves the quality of awakening. Both electroacupuncture and acupuncture require acupuncture needles to be inserted into the skin and their interventions are similar in nature, so the interventions included in this study were both of them. It is noteworthy that in both electroacupuncture and acupuncture they intervened 20–30 min before the start of surgery. This coincides with the timing of perioperative acupuncture application in recent years. For the selection of acupuncture points, most of the studies took PC6. This may suggest that stimulation of PC6 promotes the patient’s awakening after general anesthesia.

Electroacupuncture, a technique that applies electrical stimulation through acupuncture needles, can activate different neurotransmitters in the brain based on the frequency of stimulation. Specifically, electroacupuncture administered at a frequency of 2/100 Hz can stimulate the release of three substances—endorphins, enkephalins, and prednisolone—in the spinal cord (10). This may also explain why the frequency of electroacupuncture in the literature included in this study is mostly 2/100 Hz.

For both papers involving POCD, postoperative MMSE scores declined in both the intervention and control groups compared to their preoperative levels, with the difference being apparent on the third postoperative day. The invasive acupuncture group had higher scores than control group. However, the impact of cognitive scores over a longer postoperative period is lacking and more high-quality, large sample size clinical studies are needed to add to the evidence.

It is important to acknowledge the potential side effects associated with acupuncture, considering its invasive nature. While bleeding stands as the most commonly reported adverse reaction, rare occurrences of pneumothorax (11), hemothorax (12), and even cases like the one documented by Abe Daishiro (13), involving vertebral artery perforation due to fractured and displaced silver needles, emphasize the significance of proper training for acupuncture practitioners. The scarcity of experienced acupuncturists remains a notable concern raised by critics in surveys gauging the sentiments of healthcare professionals towards acupuncture in Australia (14).

Nevertheless, patients’ enthusiasm for perioperative acupuncture remains undiminished. In a preoperative assessment survey conducted at the Mayo Clinic (15), a remarkable 68.4% of participants expressed keen interest in receiving acupuncture during the perioperative period. Notably, the approval rate for complimentary acupuncture services reached an impressive 86.7%, underscoring the substantial potential for the perioperative application of acupuncture in the Western world. This view was confirmed across the pond in European. Acupuncture is widely available and promoted in Switzerland and France. Switzerland’s insurance is the most supportive of acupuncture in all of European, with reimbursement rates of up to 70–80%, and if the acupuncturist is a licensed Western medical practitioner, the patient can receive an even higher reimbursement rate. In France, due to the large number of acupuncture clinics, the competition between clinics has led directly to lower prices for acupuncture. It is easy to see how the low cost could greatly increase the acceptance of acupuncture by patients in the European and American countries.

Owing to linguistic barriers and other pertinent factors, the comprehensive adoption of traditional Chinese acupuncture in the Western world poses considerable challenges. Consequently, a process of localization has emerged, wherein dry needling therapy and battlefield acupuncture (16, 17) offer expedient and efficient means of attaining analgesic effects. As a result, these approaches find extensive utilization in the realms of combatting opioid abuse and managing pain in military settings.

### 4.1 Analysis of included literature

At the same time the subject of this literature is the awakening process after general anesthesia, and patients with non-general anesthesia do not exclude. The two papers included in this study had TIVA anesthesia although there was an anesthetic awakening process, but there was no use of inhalational anesthetics, which may increase the heterogeneity of the study when compared to anesthesia with a combination of sedation and suction and the difference in the type of intervention.

Of the types of surgeries included in these 18 articles in the literature, 7 were on the digestive system, which accounted for the largest proportion. Thoracic surgeries were next in importance with 5 articles. However, urological surgeries gynecological surgeries orthopedic surgeries neurological surgeries accounted for a lesser percentage. The patient population for these surgeries were all adults, so the findings of this study cannot be extended to the minor patient population.

There was variation in the neurostimulation instrumentation utilized within the electroacupuncture group, further contributing to heterogeneity. The absence of allocation concealment methods presents a direct risk to the research due to inadequate attention. Additionally, only some of the studies reported using blinding methods, which increases the risk of measurement bias. The absence of allocation concealment methods poses a direct risk to the research due to lack of attention.

The combined analysis of time to open eyes, time to extubation, and immediate heart rate at extubation demonstrated significant heterogeneity. To explore potential sources of this heterogeneity, we performed subgroup analyses for time to open eyes and time to extubation, along with sensitivity analyses for immediate heart rate at extubation. For the time to open eyes, subgroup stratification was based on age (≤60 years and >60 years), while for time to extubation, stratification was based on the type of surgery (minor or major). The findings revealed that heterogeneity for time to open eyes was 91% in the ≤60 years group and 0% in the >60 years group, suggesting that the younger age group (≤60 years) was likely responsible for the observed heterogeneity.

Extubation time was analyzed by categorizing surgeries into minor and major groups based on duration, with minor surgeries defined as lasting ≤2 h and major surgeries as lasting >2 h. The synthesized results demonstrated significant heterogeneity between the two groups, suggesting that surgery duration was not a contributing factor to the observed heterogeneity in extubation time. While subgroup analyses of time to open eyes and extubation time revealed considerable heterogeneity, the direction of the effect size was consistent across all groups. This consistency indicates that invasive acupuncture facilitates postoperative awakening and extubation in both minor and major surgeries, regardless of age group (≤60 years or >60 years).

Subgroup analyses demonstrated a reduction in heterogeneity across all subgroups, indicating that subgroup characteristics may contribute as a potential risk factor for the observed heterogeneity. However, this suggests that subgroup information alone is not the sole determinant of heterogeneity.

In the synthesized analysis of immediate heart rate at extubation, we observed that excluding one study (Yan, 2021) reduced the heterogeneity among the remaining three studies to 0%. This finding suggests that the Yan (2021) study contributed significantly to the observed heterogeneity. This may be explained by the fact that, in Yan’s (2021) study, epidural analgesia was administered intraoperatively, effectively reducing the patients’ pain response during the immediate extubation period. Since pain is a significant contributor to increased heart rate in the postoperative period, this intervention likely influenced the observed outcomes. Additionally, epidural anesthesia can slow heart rate if the level of anesthesia is excessively high (above T4), as it may block the cardiac sympathetic nerves.

The conclusions of the study should be viewed with caution due to the possible heterogeneity of the synthesized results. Future clinical studies of acupuncture should aim for multicenter, long follow-up periods, and standardized treatment protocols.

## Data Availability

The datasets presented in this study can be found in online repositories. The names of the repository/repositories and accession number(s) can be found in the article/Supplementary material.
