# Supplementary material for: Effect of invasive acupuncture on awakening quality after general anesthesia: systematic review and meta-analysis
Source: Front Med (Lausanne). 2025 Jan 13;11:1502619. doi: 10.3389/fmed.2024.1502619 (PMC11770014; doi:10.3389/fmed.2024.1502619)

PubMed search strategy:

| Step | Content |
| --- | --- |
| 1 | Search: general anesthesia |
| 2 | Search: ((awakening) OR (emergence)) OR (recovery) |
| 3 | Search: eye-opening |
| 4 | Search: extubation |
| 5 | Search: PACU |
| 6 | Search: ((acupoint) OR (acupuncture)) OR (electroacupuncture) |
| 7 | Search: randomized controlled trial |
| 8 | Search: ((((((general anesthesia) AND (((awakening) OR (emergence)) OR (recovery))) OR (eye-opening)) OR (extubation)) OR (PACU)) AND (((acupoint) OR (acupuncture)) OR (electroacupuncture))) AND (randomized controlled trial) |

CNKI ([中国知网 (cnki.net)](https://www.cnki.net/)) search strategy:


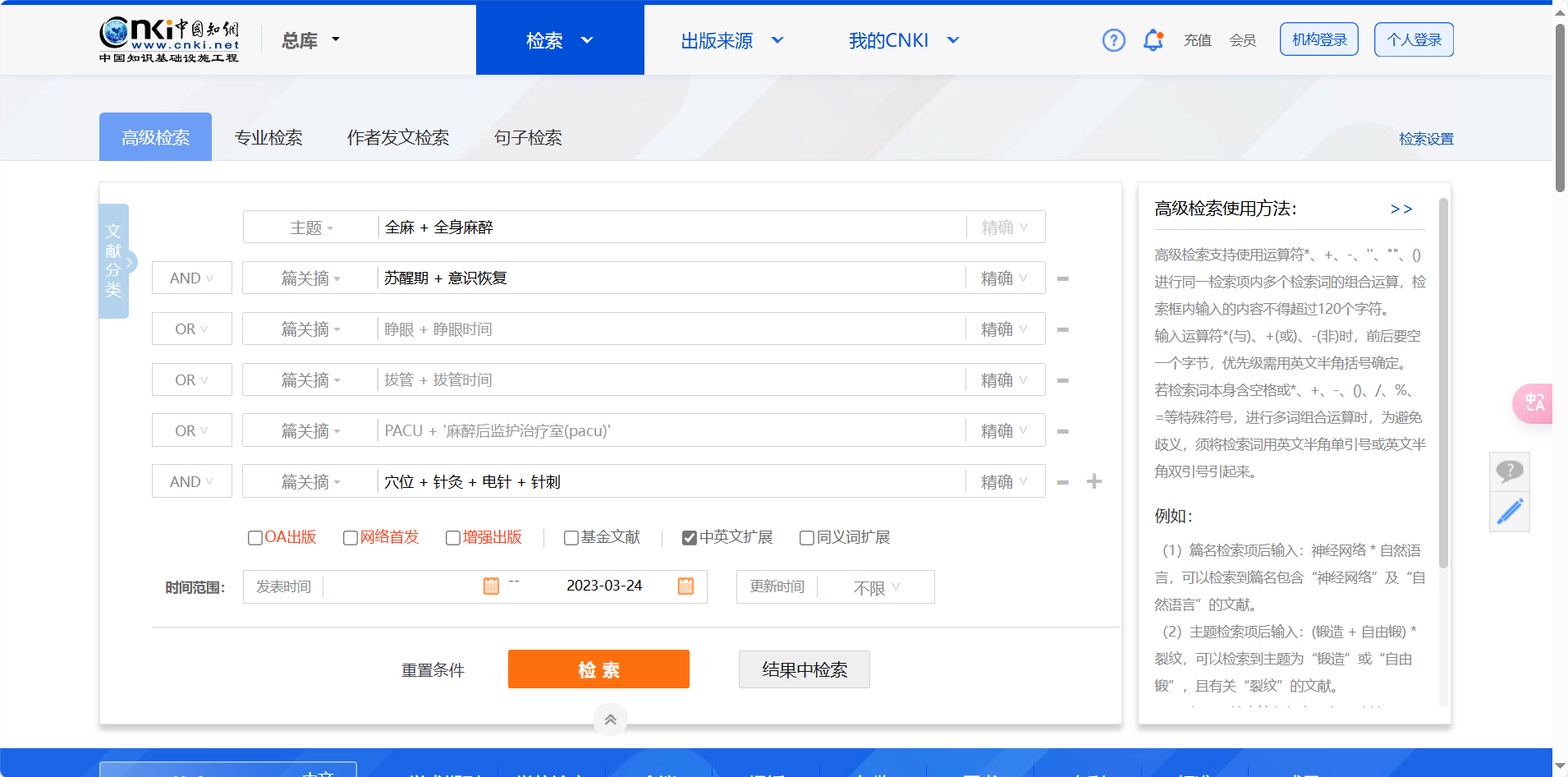


The filter is “research paper”.


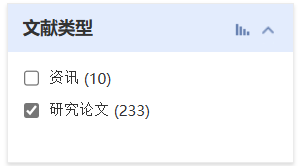

Supplement: Supplementary file 3 [file Data_Sheet_3.DOCX]
