# Supplementary material for: Effect of invasive acupuncture on awakening quality after general anesthesia: systematic review and meta-analysis
Source: Front Med (Lausanne). 2025 Jan 13;11:1502619. doi: 10.3389/fmed.2024.1502619 (PMC11770014; doi:10.3389/fmed.2024.1502619)
Supplement: Supplementary file 5 [file Data_Sheet_5.DOCX]

| **Invasive acupuncture group compared to Control group for quality of awakening after general anesthesia** | | | | | | |
| --- | --- | --- | --- | --- | --- | --- |
| **Patient or population:** patients with quality of awakening after general anesthesia **Settings:**  **Intervention:** Invasive acupuncture group **Comparison:** Control group | | | | | | |
| **Outcomes** | **Illustrative comparative risks* (95% CI)** | | **Relative effect (95% CI)** | **No of Participants (studies)** | **Quality of the evidence (GRADE)** | **Comments** |
|  | Assumed risk | Corresponding risk |  |  |  |  |
|  | **Control group** | **Invasive acupuncture group** |  |  |  |  |
| **Time to open eyes** |  | The mean time to open eyes in the intervention groups was **6.42 lower** (8.17 to 4.66 lower) |  | 677 (10 studies) | ⊕⊕⊝⊝ **low**^1^ |  |
| **Time to extubation** |  | The mean time to extubation in the intervention groups was **5.84 lower** (8.12 to 3.56 lower) |  | 745 (12 studies) | ⊕⊕⊝⊝ **low**^2^ |  |
| **MAP immediately after extubation** |  | The mean map immediately after extubation in the intervention groups was **18.54 lower** (22.69 to 14.39 lower) |  | 160 (3 studies) | ⊕⊕⊝⊝ **low**^3,4^ |  |
| **HR immediately after extubation** |  | The mean hr immediately after extubation in the intervention groups was **14.85 lower** (23.9 to 5.81 lower) |  | 184 (4 studies) | ⊕⊕⊝⊝ **low**^2^ |  |
| **Incidence of POCD** | **Study population** | | **OR 0.56**  (0.28 to 1.11) | 158 (2 studies) | ⊕⊕⊕⊕ **high** |  |
|  | **359 per 1000** | **239 per 1000** (136 to 383) |  |  |  |  |
|  | **Moderate** | |  |  |  |  |
|  | **359 per 1000** | **239 per 1000** (136 to 383) |  |  |  |  |
| **Incidence of agitation** | **Study population** | | **OR 0.42**  (0.11 to 1.65) | 220 (3 studies) | ⊕⊕⊕⊕ **high** |  |
|  | **55 per 1000** | **24 per 1000** (6 to 87) |  |  |  |  |
|  | **Moderate** | |  |  |  |  |
|  | **50 per 1000** | **22 per 1000** (6 to 80) |  |  |  |  |
| *The basis for the **assumed risk** (e.g. the median control group risk across studies) is provided in footnotes. The **corresponding risk** (and its 95% confidence interval) is based on the assumed risk in the comparison group and the **relative effect** of the intervention (and its 95% CI). **CI:** Confidence interval; **OR:** Odds ratio; | | | | | | |
| GRADE Working Group grades of evidence **High quality:** Further research is very unlikely to change our confidence in the estimate of effect.  **Moderate quality:** Further research is likely to have an important impact on our confidence in the estimate of effect and may change the estimate. **Low quality:** Further research is very likely to have an important impact on our confidence in the estimate of effect and is likely to change the estimate. **Very low quality:** We are very uncertain about the estimate. | | | | | | |
| ^1^ I2 = 90% ^2^ I2 = 93% ^3^ I2 = 56% ^4^ Egger test P = 0.002 | | | | | | |
