# Supplementary material for: Effect of invasive acupuncture on awakening quality after general anesthesia: systematic review and meta-analysis
Source: Front Med (Lausanne). 2025 Jan 13;11:1502619. doi: 10.3389/fmed.2024.1502619 (PMC11770014; doi:10.3389/fmed.2024.1502619)
Supplement: Supplementary file 6 [file Data_Sheet_6.DOCX]

**Funnel plot:**

**Funnel plot of eye-opening time**


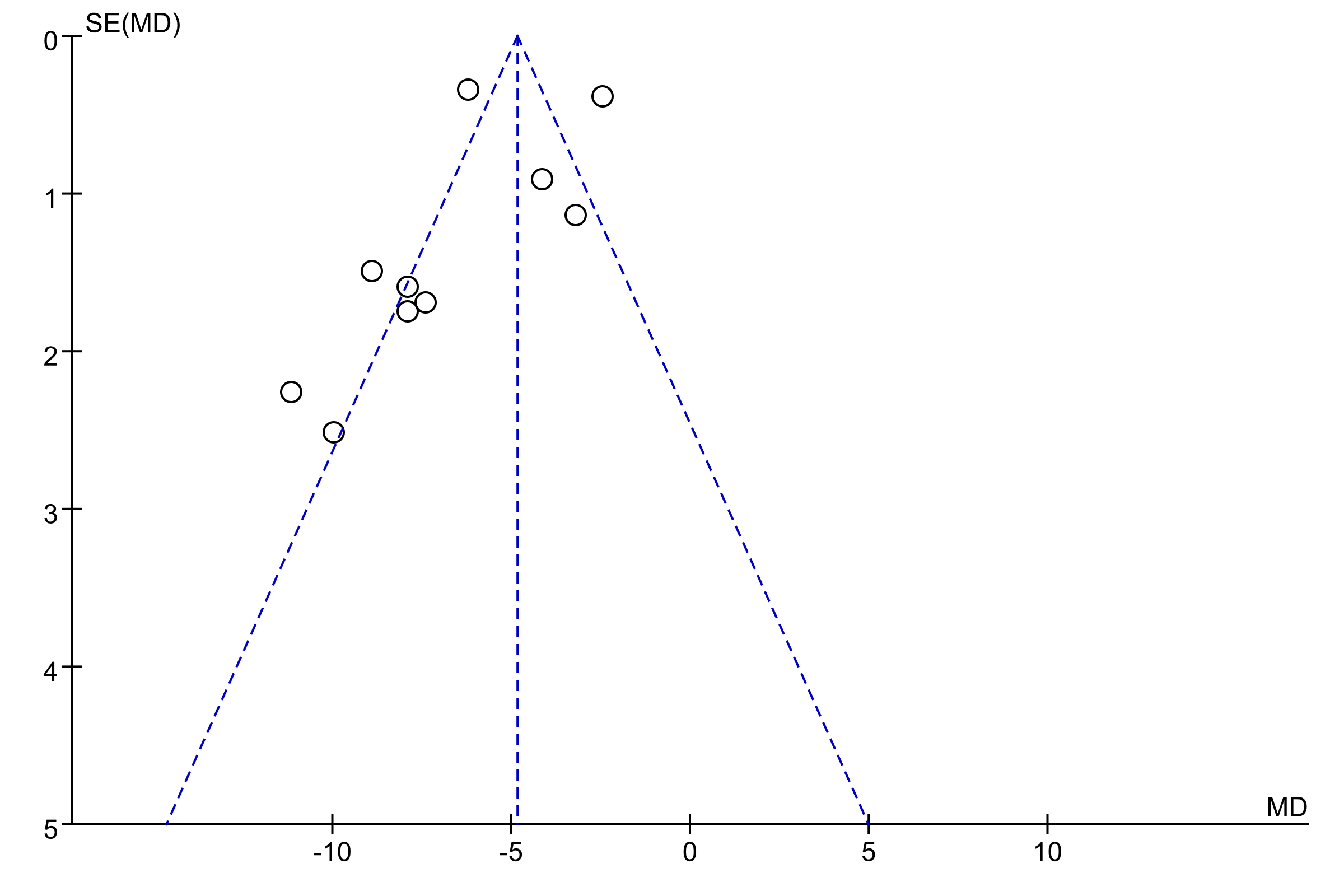


The dots in the line are generally balanced, no publication bias exists.

**Funnel plot of extubation time**


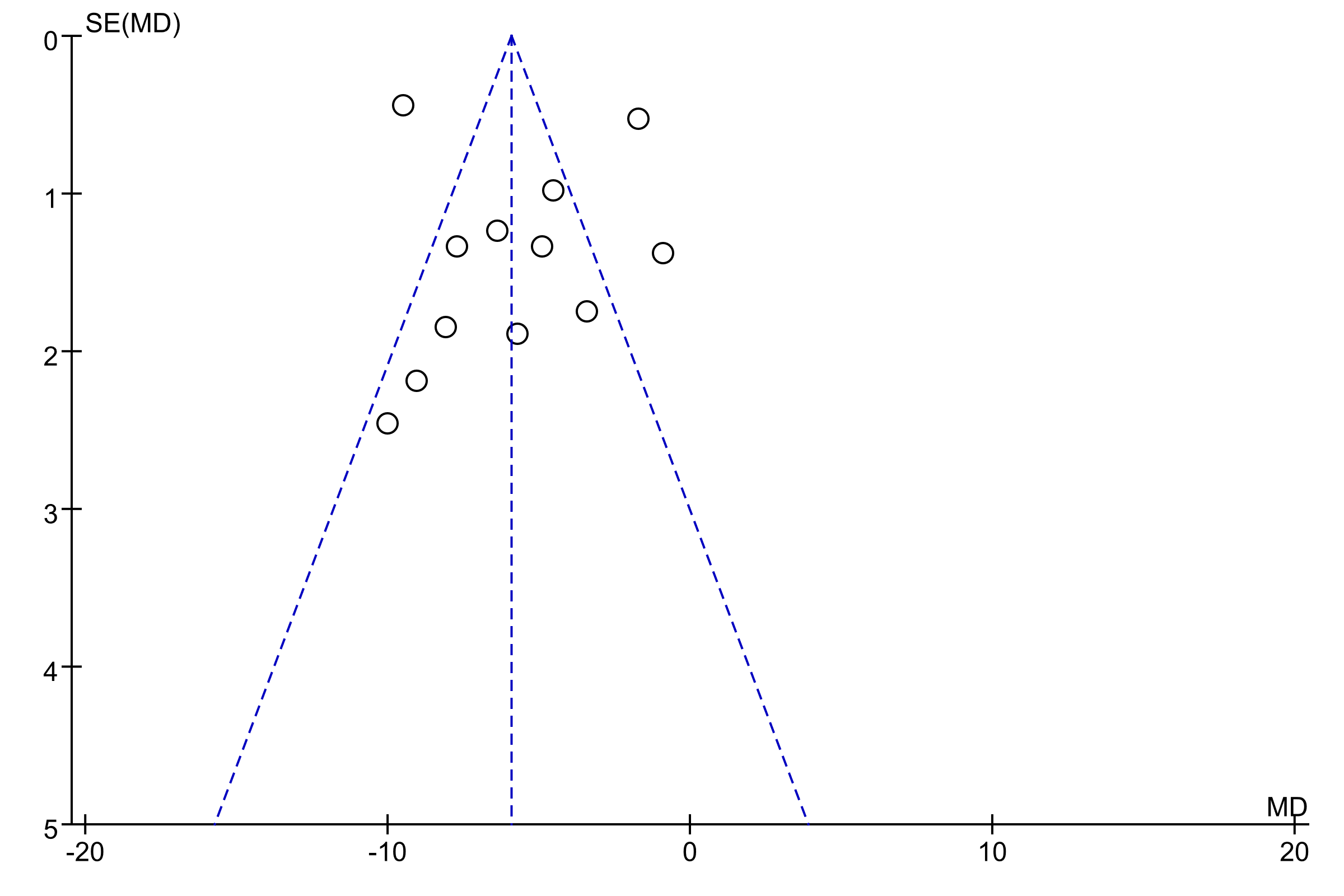


The dots in the line are generally balanced, no publication bias exists.

**Egger test:**

**MAP immediately after extubation**
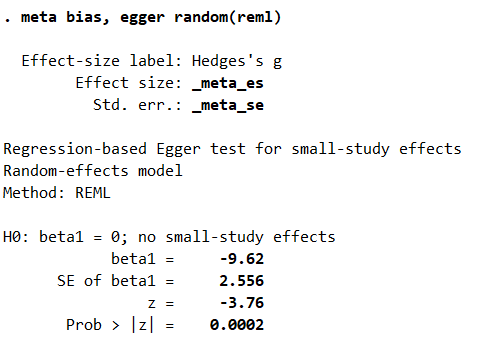


P = 0.002, exists publication bias exists.

**HR immediately after extubation:**


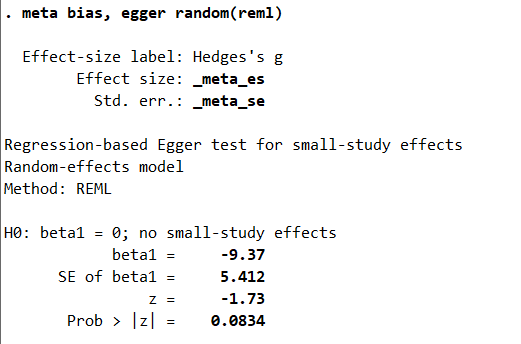


P = 0.0834, no publication bias exists.

Incidence of POCD


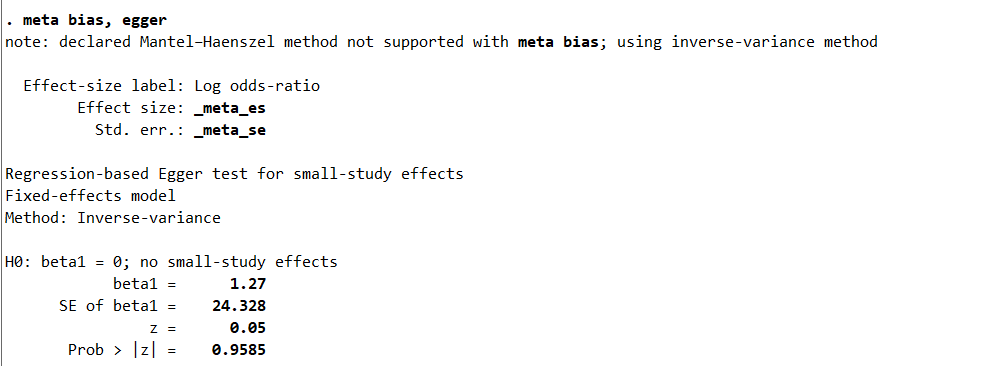


P = 0.9585, no publication bias exists.

Incidence of agitation


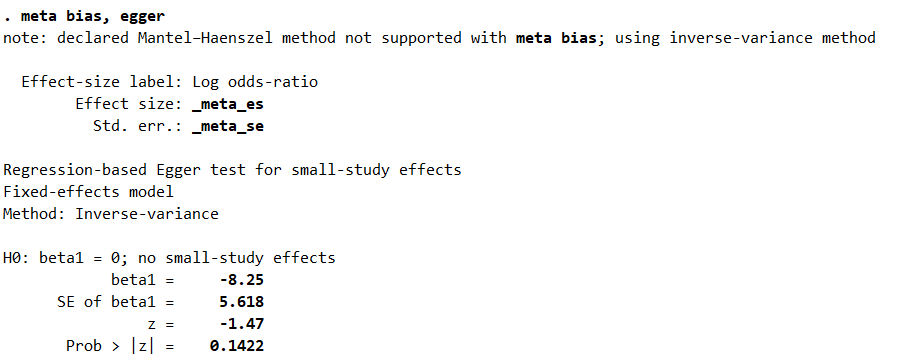


P = 0.1422, no publication bias exists.
